# Supplementary material for: Rif1 restrains the rate of replication origin firing in Xenopus laevis
Source: Commun Biol. 2023 Jul 29;6:788. doi: 10.1038/s42003-023-05172-8 (PMC10387115; doi:10.1038/s42003-023-05172-8)
Supplement: Supplementary file 2 — Supplementary Informations [file 42003_2023_5172_MOESM2_ESM.pdf]

Rif1 restrains the rate of replication origin firing in *Xenopus laevis*

Olivier Haccard[1], Diletta Ciardo[2], Hemalatha Narrissamprakash[1], Odile Bronchain[3], Akiko Kumagai[4], William G. Dunphy[4], Arach Goldar[1], Kathrin Marheineke*[1]

Supplementary Information

Supplementary Figure 1: Strong increase of replication extent after Rif1 depletion.

Supplementary Figure 2: Non-homogenous organisation of replication tracks on combed fibers after mock and Rif1 depletion.

Supplementary Figure 3: Replication track patterns differ in the presence and absence of Rif1.

Supplementary Figure 4: Modeling experimental combing data with MM4

Supplementary Figure 5: Rif1 depletion increases replication foci number.

Supplementary Figure 6: Rif1 depletion increases limiting replication factors and p-MTBP, p-Treslin on chromatin.

Supplementary Figure 7: MTBP protein levels decrease after MBT during early *Xenopus laevis* development.

Supplementary Figure 8: Images of uncropped Western blots.

Supplementary Table 1: DNA combing data summary after Rif1 depletion.

Supplementary Methods 1: list of antibodies

Supplementary Methods 2: Definitions of replication parameters for DNA combing analysis

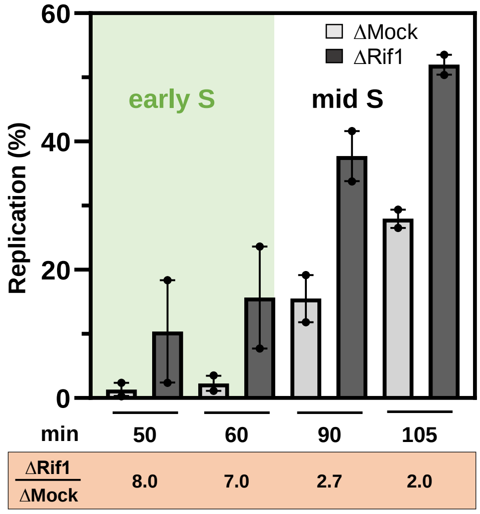

Supplementary Figure 1: Strong increase of replication extent after Rif1 depletion. Rif1 was immunodepleted from egg extracts, DNA was isolated at the indicated times, then DNA combing was performed in two independent experiments, replication extent mean with SEM (n=2) , and ratios ΔRif1/Mock were calculated.

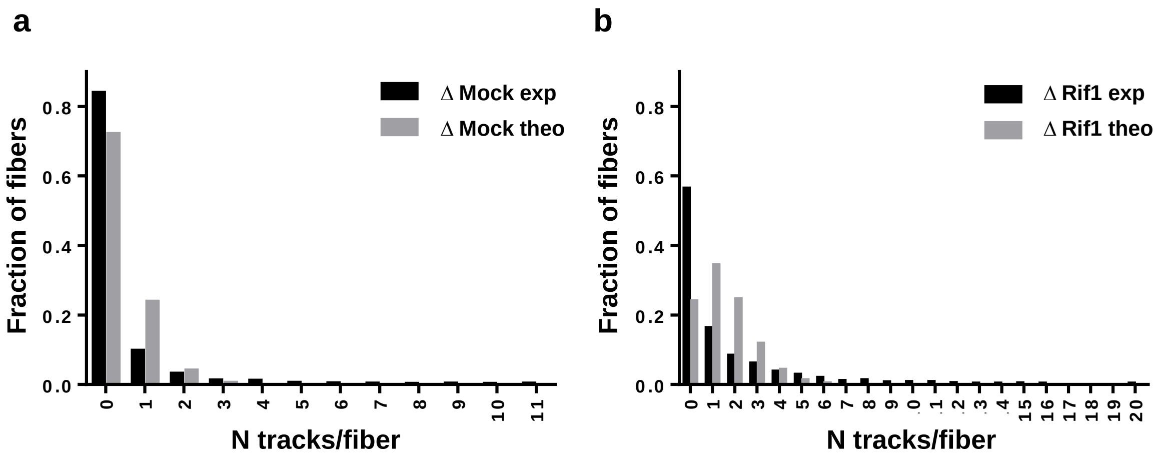

Supplementary Figure 2: Non-homogenous organisation of replication tracks on combed fibers after mock and Rif1 depletion.

Using the dataset from Fig. 2d, frequency of combed fibers versus the number of replication tracks per fiber (N) for early S phase time points (exp) compared to a Poisson distribution (random) with identical mean (theo). **a** From mock depleted extracts, Chi-squared test ($p = 1.4x10^{-164}$). **b** From Rif1 depleted extracts, Chi-squared test, p = 0.

a

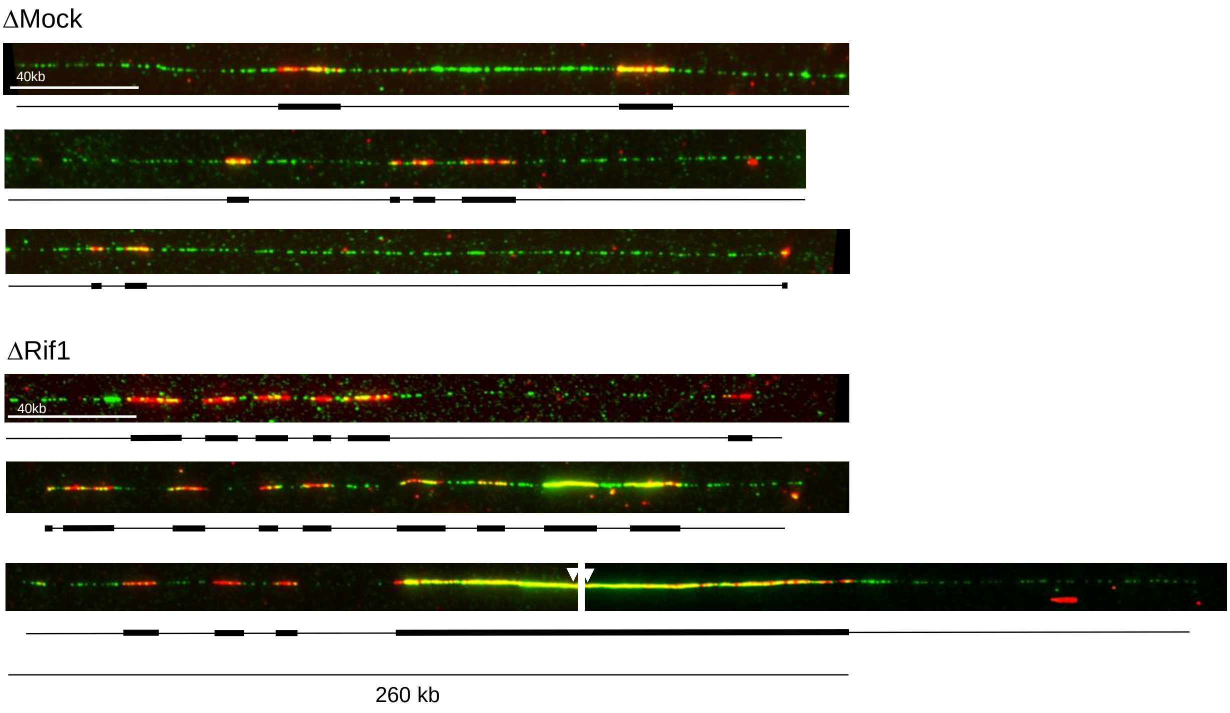

b

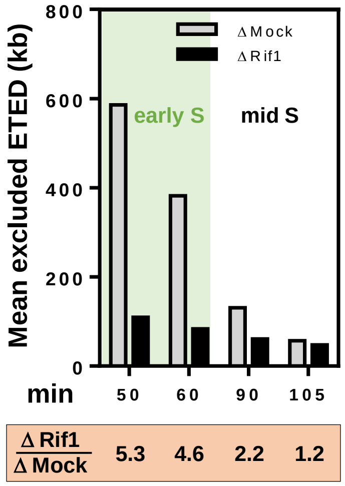

Supplementary Figure 3: Replication track patterns differ in the presence and absence of Rif1.
a Example of combed fibers with DNA (green) and replication eyes (red) from Fig. 2d, early time points. Positions of replication tracks indicated below each fiber in bold black. Third fiber in Rif1 depleted conditions is a composed fiber of two consecutive microscope fields. **b** Mean excluded eye-to-eye distances (mean excluded ETED) from mock and Rif1 depleted extracts of experiment1; corresponding ratios are indicated below.
Mean excluded ETED = excluded DNA/((N of forks/2) - N of ETED);
excluded DNA = tot DNA - (mean ETED x N of ETED).

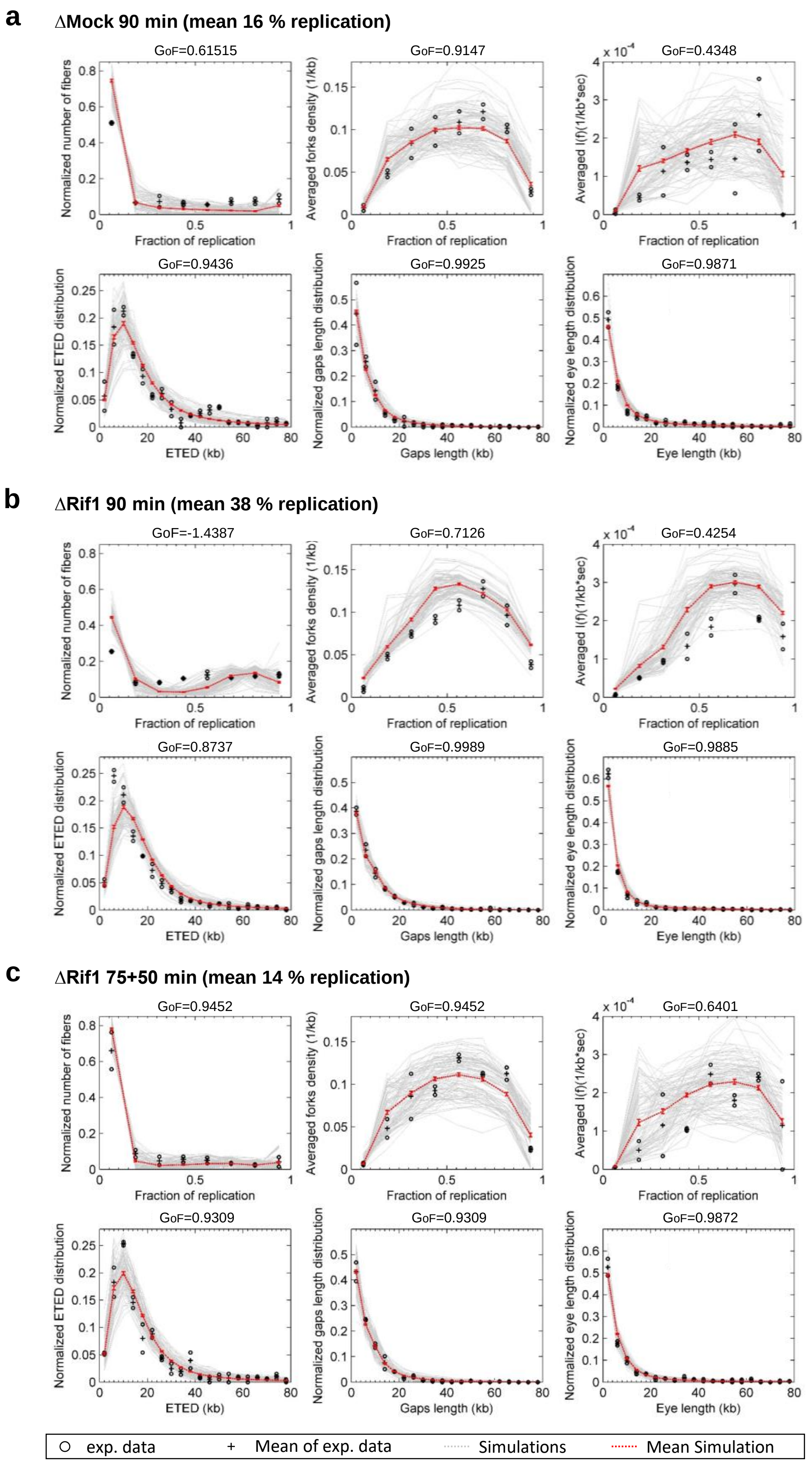

Supplementary Figure 4: Modeling experimental combing data with MM4[41]. The potential origins are heterogeneously distributed along the genome. Open circles represent normalized distribution of mean experimental values for indicated replication parameters for the two replicates (for n see Suppl. Table 1), cross represents the means for the 2 replicates. The red dashed line is the average of n = 100 independent fits (grey dashed lines) from MM4[41]. The red error bars indicate standard deviations. **a** Mock depletion, 90 min from two biological replicates, mean 16 % replication. **b** Rif1 depletion, 90 min from two biological replicates, mean 38 % replication. **c** Rif depletion, mean 14 % replication, from two biological replicates: respectively 50 min and 75 min.

The goodness of the fit (GoF) is calculated as $GoF = 1 - \frac{\|y_{fit} - y_{exp}\|^2}{\|y_{exp} - \langle y_{exp} \rangle\|^2}$ and can vary from -∞ (bad fit) to 1 (perfect fit).

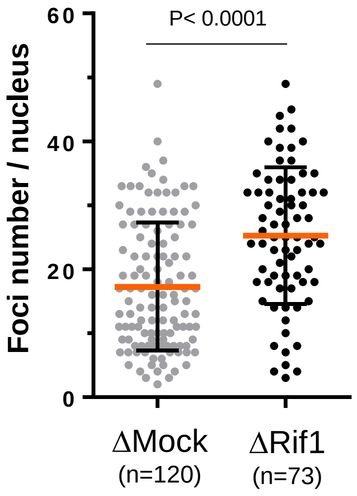

Supplementary Figure 5: Rif1 depletion increases replication foci number. As in Figure 3d, quantification of second biological replicate, mean with SD, two-tailed Mann-Whitney test.

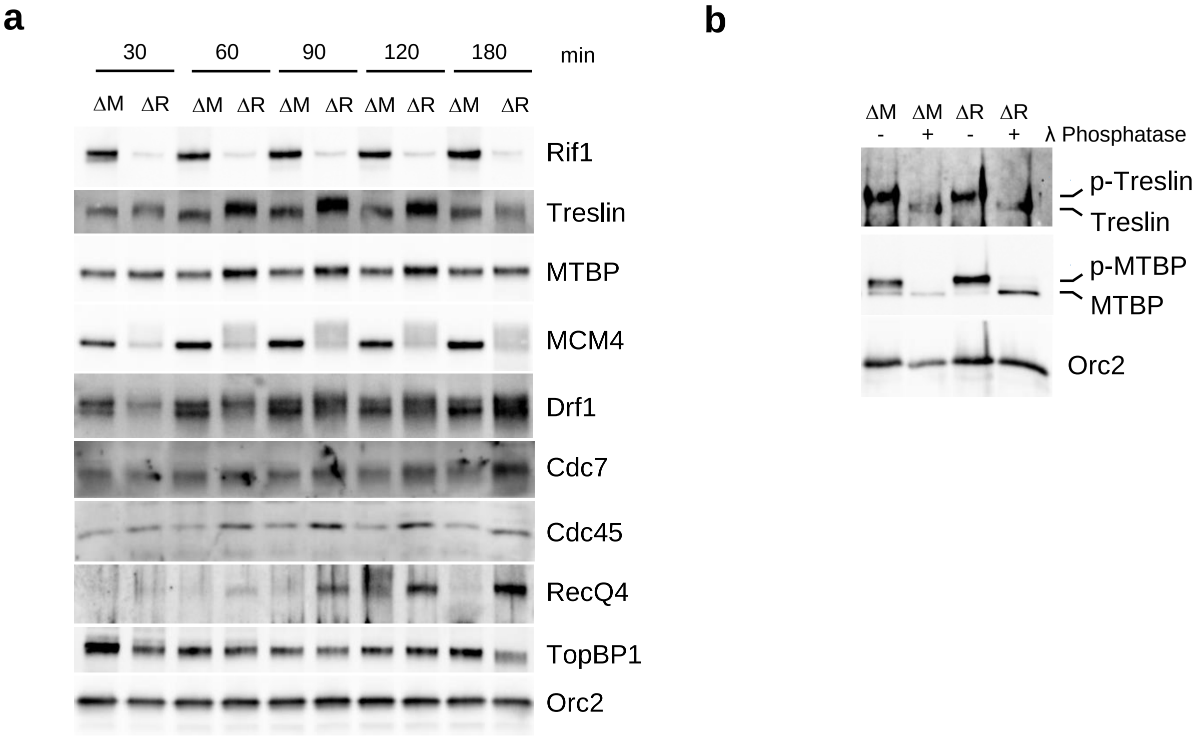

Supplementary Figure 6: Rif1 depletion increases limiting replication factors and p-MTBP, p-Treslin on chromatin.

a Chromatin fractions after Rif1 depletion or Mock depletion were isolated at different times and analyzed by western blot for indicated proteins, second biological replicate of time course experiment as in Fig. 5. **b** samples from Fig. 5b, treated with λ-phosphatase.

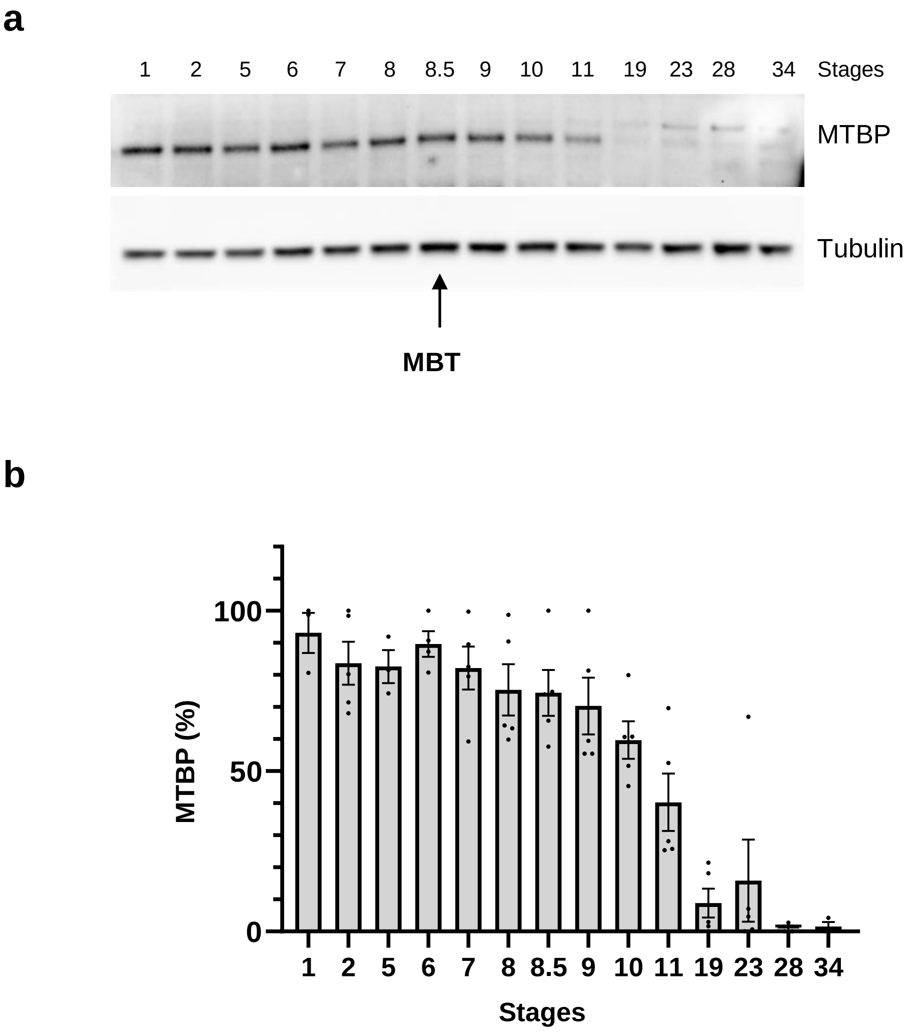

Supplementary Figure 7: MTBP protein levels decrease after MBT during early *Xenopus laevis* development.

a Time course analysis of MTBP expression throughout development, whole embryo protein extracts were analysed by western blotting against indicated proteins, tubulin was used as a loading control; MBT (mid-blastula transition). **b** quantification of MTBP abundance; three biological replicates and two technical replicates of western blot series of embryonic whole cell extracts were quantified and Rif1 abundance was plotted as mean OD normalized to Tubulin, scaled using min and max: xscaled = (x - xmin) / (xmax - xmin), Mean with SED, n = 5.

Supplementary Figure 8: Images of uncropped Western blots.

Uncropped immunoblots used in Figure 1a

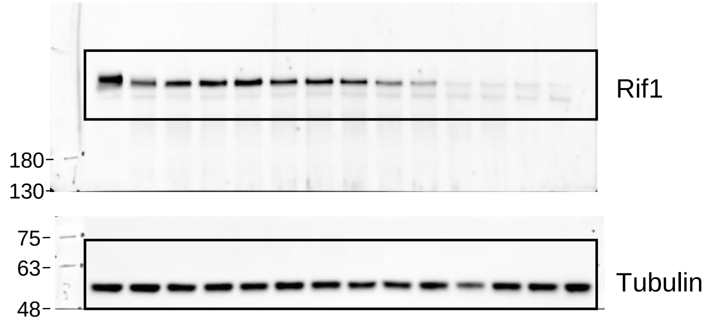

Uncropped immunoblots used in Figure 1c

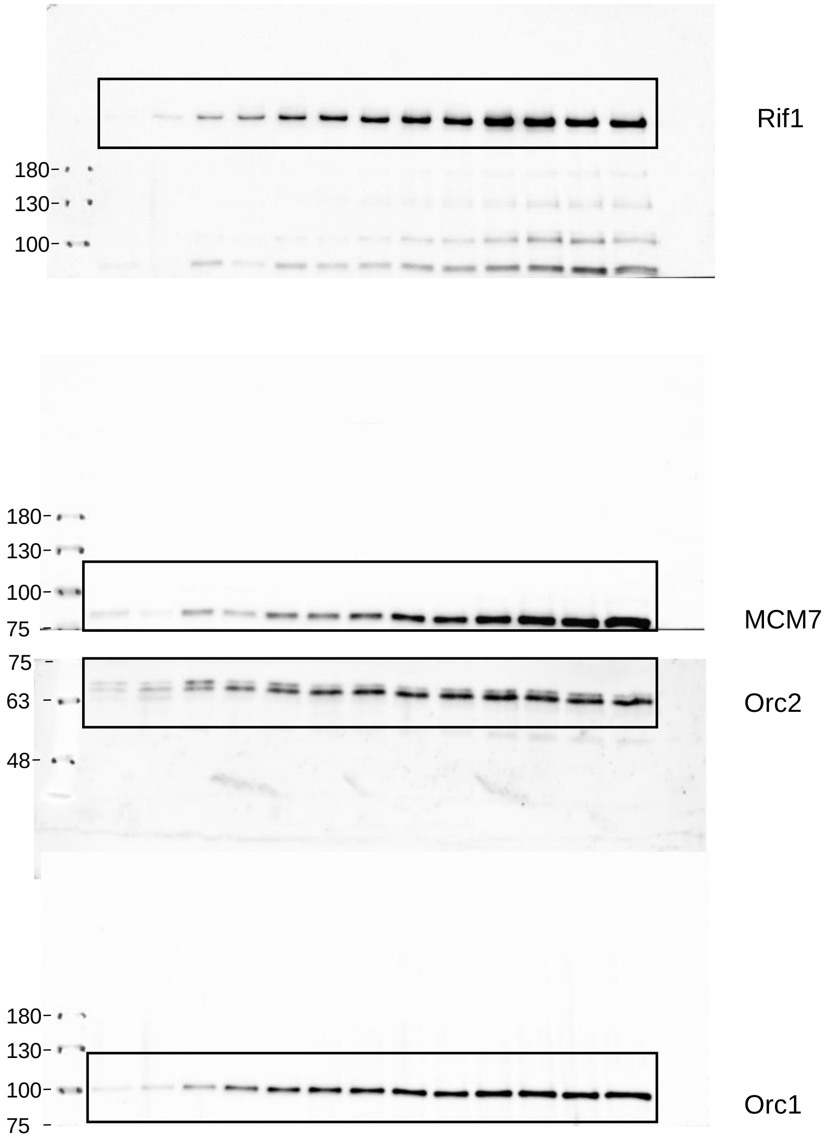

Supplementary Figure 8 (continued)

Uncropped immunoblots used in Figure 5b

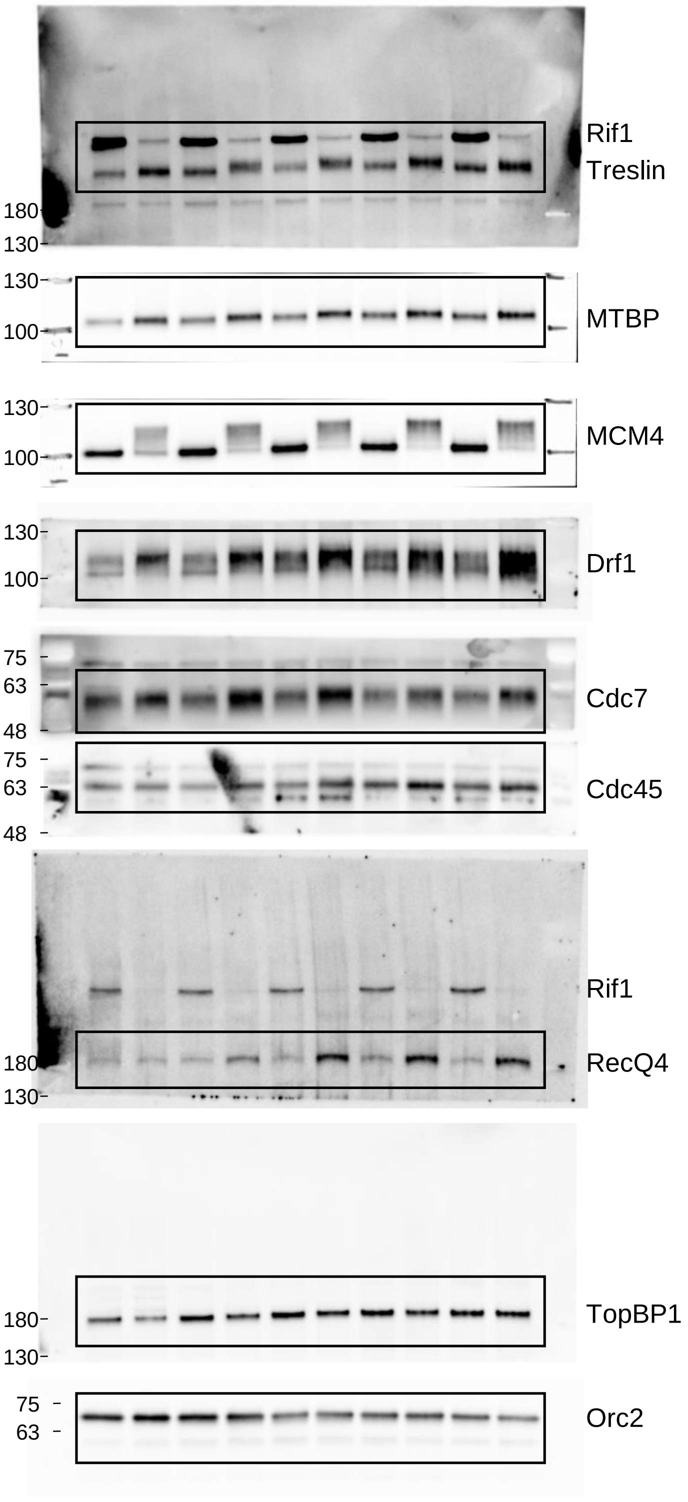

Uncropped immunoblots used in Figure 5d

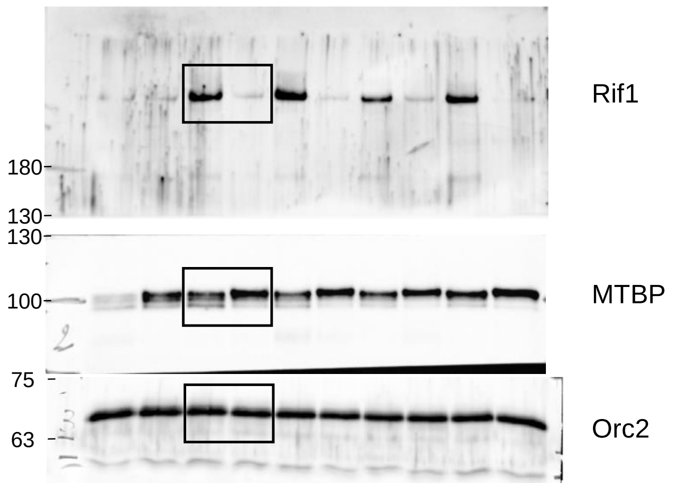

Supplementary Figure 8 (continued)

Uncropped immunoblots used in Supplementary Figure 6a

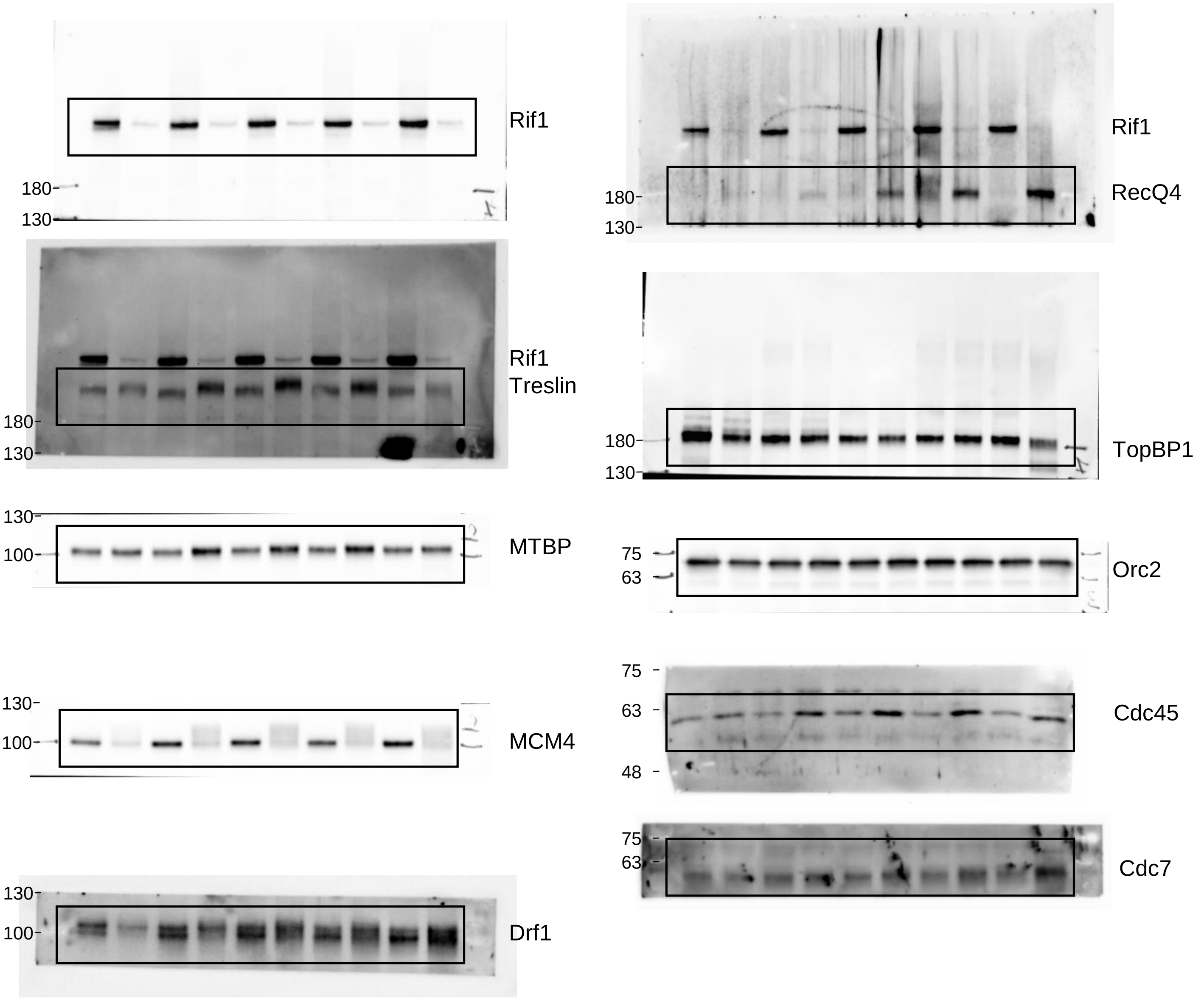

Uncropped immunoblots used in Supplementary Figure 6b

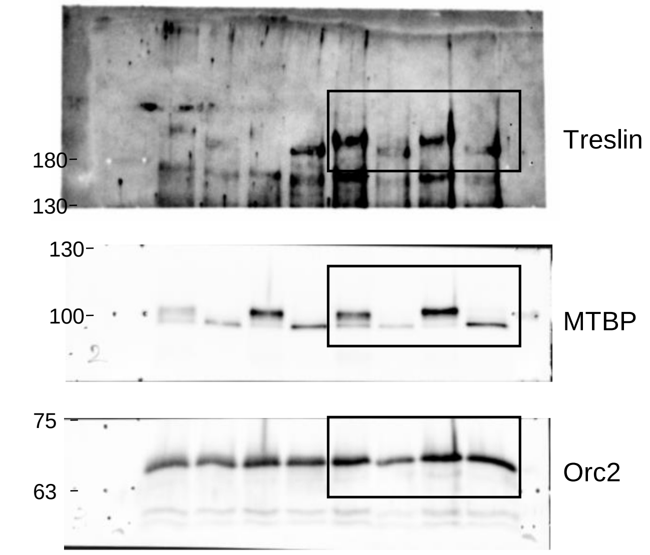

Uncropped immunoblots used in Supplementary Figure 7a

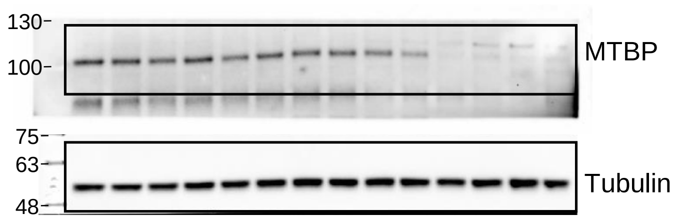

Supplementary Table 1: DNA combing data summary after Rif1 depletion.

Replicate 1	early S phase						mid S phase				
Min	50 min		60 min		75 min		90 min		105 min		sum total
condition	ΔMock	ΔRif1	ΔMock	ΔRif1	ΔMock	ΔRif1	ΔMock	ΔRif1	ΔMock	ΔRif1	
Analysed DNA (kb)	57317.4	33913.6	50565.6	63104.9	34450.7	55022.6	32863.5	22546.7	24290.0	34122.3	408197.1
Replicated DNA(kb)	1376.3	6234.0	1778.9	14876.4	2705.7	18221.1	6299.0	9372.4	7151.3	17207.5	85222.5
**Replication Extent**	**0.024**	**0.184**	**0.035**	**0.236**	**0.079**	**0.331**	**0.192**	**0.416**	**0.294**	**0.504**	
**Number of analysed fibers**	**482**	**352**	**673**	**690**	**366**	**652**	**288**	**258**	**352**	**349**	**4462.0**
Number of fully replicated fibers	2	15	6	29	33	9	11	14	34	15	
Number of unreplicated Fibers	393	133	548	186	171	117	111	54	52	41	
fraction of unreplicated fibers	0.82	0.38	0.81	0.27	0.47	0.18	0.39	0.21	0.15	0.12	
mean size of all fibers (kb)	118.9	96.3	75.1	91.5	94.1	84.4	114.1	87.4	69.0	97.8	91.5
mean size of unreplicated fibers (kb)	117.3	91.8	71.0	85.9	106.1	72.3	129.0	83.7	85.6	95.7	
**Number of replication eyes**	161	503	196	1112	226	1639	481	652	558	1247	6775.0
**Mean Eye Length (kb)**	**6.3**	**8.5**	**6.7**	**9.6**	**6.8**	**9.1**	**10.7**	**10.4**	**8.7**	**10.1**	
**Number of eye to eye distances (ETED)**	82	341	101	737	125	1193	353	497	379	1000	4808.0
**Mean ETED length (kb)**	**23.6**	**19.4**	**22.3**	**21.6**	**25.2**	**18.4**	**22.1**	**16.3**	**19.1**	**15.7**	
**Mean excluded ETED length (kb)**	**586.1**	**109.6**	**381.9**	**83.8**	**182.5**	**53.3**	**130.8**	**60.7**	**56.9**	**47.3**	
**Number of replication forks**	353	1180	455	2600	593	3628	1089	1469	1357	2779	15503.0
**Fork Density (forks/100 kb)**	**0.62**	**3.48**	**0.90**	**4.12**	**1.72**	**6.59**	**3.31**	**6.52**	**5.59**	**8.14**	
**Rif/Mock FD**	5.65		4.58		3.83		1.97		1.46		
**Number of gaps**	192	677	259	1488	367	1989	608	817	799	1532	8728.0
Mean gap size (kb)	7.2	9.2	6.9	10.0	7.4	9.2	10.4	11.5	9.0	11.2	

**Replicate 2**	early S phase						mid S phase				
min	**50 min**		**60 min**		**75 min**		**90 min**		**105 min**		**sum total**
Condition	**ΔMock**	**ΔRif1**	**ΔMock**	**ΔRif1**	**ΔMock**	**ΔRif1**	**ΔMock**	**ΔRif1**	**ΔMock**	**ΔRif1**	
Analysed DNA (kb)	29676.5	38244.1	47274.0	32949.2	24942.5	38001.2	40709.9	51519.9	47109.9	50042.1	400469.2
Replicated DNA(kb)	103.2	920.3	527.4	2552.9	964.2	3853.5	4824.0	17415.5	12500.3	26768.7	
**Replication Extent**	**0.003**	**0.024**	**0.011**	**0.077**	**0.039**	**0.101**	**0.118**	**0.338**	**0.265**	**0.535**	
**Number of analysed fibers**	**312**	**457**	**371**	**402**	**352**	**525**	**675**	**917**	**780**	**1320**	**6111.0**
Number of fully replicated fibers	0	3	1	23	4	5	56	78	152	367	
Number of unreplicated Fibers	267	374	307	218	264	337	220	143	147	116	
fraction of unreplicated fibers	0.86	0.82	0.83	0.54	0.75	0.64	0.33	0.16	0.19	0.09	
Mean size of all fibers (kb)	95.1	83.7	127.4	82.0	70.9	72.4	60.3	56.2	60.4	37.9	65.5325086
Mean size of unreplicated fibers (kb)	88.2	79.4	127.3	89.8	70.0	69.6	73.2	61.0	75.7	54.7	
Number of replication eyes	41	111	114	273	116	396	447	1296	1025	1400	5219.0
**Mean Eye Length (kb)**	**1.8**	**5.8**	**4.0**	**6.5**	**4.9**	**7.3**	**5.7**	**8.7**	**7.5**	**10.0**	
Number of eye to eye distances (ETED)	5	53	55	161	64	251	202	839	701	953	3284.0
**Mean ETED length (kb)**	**35.1**	**25.5**	**29.7**	**14.6**	**14.3**	**15.7**	**24.7**	**16.7**	**16.4**	**15.2**	
Number of replication forks	92	266	241	692	297	922	1215	3268	2627	4067	13687.0
Mean excluded ETED (kb)	nd	nd	696.8	165.4	284.3	162.1	88.1	47.1	58.2	32.92	
**Fork Density (forks/100 kb)**	**0.31**	**0.70**	**0.51**	**2.10**	**1.19**	**2.43**	**2.98**	**6.34**	**5.58**	**8.13**	
**Rif/mock FD**	2.24		4.12		2.04		2.13		1.46		
**Number of gaps**	51	155	127	419	181	526	768	1972	1602	2667	8468.0
mean gap length (kb)	2.0	5.9	4.2	6.1	5.3	7.3	6.3	8.8	7.8	10.0	

Supplementary Methods 1

List of antibodies used in this study

Antigen	Host	Source	Reference	Application	RRID and/or Doi
**Primary antibodies**					
Anti-*Xenopus* RIF1	Rabbit	Covalab, Villeurbanne, France		1:500 (IF)<br>1:1000 (WB)	10.4161/cc.11.6.19636<br>10.1093/n10.1093 /nar/gkab756<br>10.7554/eLife.75741
Anti-Xenopus and Human Treslin	Rabbit	A. Kumagai and W. G. Dunphy, Pasadena, USA		1:1000 (WB)	10.1016/j.cell.2009.12.049
Anti-*Xenopus* MTBP	Rabbit	A. Kumagai and W. G. Dunphy, Pasadena, USA		1:1000 (WB)	10.1091/mbc.E17-07-0448
Anti-human MCM4	Rabbit	Bethyl lab	A300-193A	1:2000 (WB)	AB_162720
Anti-Xenopus Drf1	Rabbit	A. Kumagai and W. G. Dunphy, Pasadena, USA		1:1000 (WB)	10.1074/jbc.M307144200
Anti-Xenopus Cdc7	Rabbit	J. Walter		1:1000 (WB)	10.1016/s1097-2765(00)80241-5
Anti-Xenopus Cdc45	Rabbit	A. Kumagai and W. G. Dunphy, Pasadena, USA		1:500 (WB)	10.1091/mbc.e05-07-0671
Anti-Xenopus RecQ4	Rabbit	A. Kumagai and W. G. Dunphy, Pasadena, USA		1:1000 (WB)	10.1016/j.cell.2005.05.015
Anti-Xenopus TopBP1	Rabbit	A. Kumagai and W. G. Dunphy, Pasadena, USA		1:1000 (WB)	10.1016/j.cell.2005.12.041
Anti-*Xenopus* ORC2	Rabbit	Gift from Dr. J. L. Maller (EXRC), Portsmouth, UK		1:1000 (WB)	10.1038/s41467-022-34779-4<br>10.15252/embj.201796585
Anti-*Xenopus* ORC1	Rabbit	Gift from R. A. Laskey, Cambridge, UK		1:1000 (WB)	10.1016/S0960-9822(96)00746-4
Anti-*Xenopus* MCM7	Rabbit	Gift from R. A. Laskey, Cambridge, UK		1:1000 (WB)	10.1073/pnas. 93.19.10189
Anti-α Tubulin	Mouse	Sigma	T5168	1:10000 (WB)	AB_477579
Anti-human ssDNA	Mouse	DSHB	autoanti-ssDNA	1:100 (comb)	AB_10805144
**Secondary antibodies**					
AlexaFluor488 anti-mouse	Rabbit	Invitrogen	A11059	1:50 (comb)	AB_2534106
AlexaFluor488 anti- rabbit	Goat	Thermo Fisher Scientific	A11008	1:200 (IF)<br>1:50 (comb)	AB_143165
Anti-streptavidin biotinylated	Mouse	Abcyss	BA-0500	1:50 (comb)	AB_2336221
AlexaFluor594 conjugated streptavidin	Mouse	Invitrogen	S11227	1:50 (comb)	
HRP anti-mouse IgG	Goat	Sigma-Aldrich	A4416	1:10000 (WB)	AB_258167
HRP anti-rabbit IgG	Donkey	GE Healthcare	NA934	1:10000 (WB)	AB_772206

If, immunofluorescence; WB, western blot; comb, DNA combing

Supplementary Methods 2

Definitions of replication parameters for DNA combing analysis

Replicated fraction of a fiber *f(t)*	Sum of all red tracks (EL) on a fiber/length of fiber (green track)
Total replicated fraction of a population of fibers (*f(t))*	Sum of all EL/ sum of fiber length
Percentage of unreplicated fibers	N of fibers with no red track/ N of all fibers
Fork density of a population of fibers	Number of forks/sum of fiber lengths
Active origin for *I(t)* calculation	EL <3 kb
Frequency of initiation *I(t)*	Number of active origins / ((1-*f(t))**fiber length*180 sec)
Excluded DNA (kb)	Total DNA - (mean ETED x N of ETED)
Mean excluded ETED (kb)	Excluded DNA/((N of forks/2) - N of ETED)
