# Supplementary material for: Rif1 restrains the rate of replication origin firing in Xenopus laevis
Source: Commun Biol. 2023 Jul 29;6:788. doi: 10.1038/s42003-023-05172-8 (PMC10387115; doi:10.1038/s42003-023-05172-8)
Supplement: Supplementary file 3 — Description of Additional Supplementary Files [file 42003_2023_5172_MOESM3_ESM.pdf]

Description of Additional Supplementary Files

File name: Supplementary Data
Description: The source data behind the graphs in the paper (excel file with tabs).
