# Supplementary material for: Rif1 restrains the rate of replication origin firing in Xenopus laevis
Source: Commun Biol. 2023 Jul 29;6:788. doi: 10.1038/s42003-023-05172-8 (PMC10387115; doi:10.1038/s42003-023-05172-8)
Supplement: Supplementary file 5 — Reporting Summary [file 42003_2023_5172_MOESM5_ESM.pdf]

nature portfolio

Corresponding author(s): Kathrin Marheineke

Last updated by author(s): Jul 18, 2023

Reporting Summary

Nature Portfolio wishes to improve the reproducibility of the work that we publish. This form provides structure for consistency and transparency in reporting. For further information on Nature Portfolio policies, see our Editorial Policies and the Editorial Policy Checklist.

Statistics

For all statistical analyses, confirm that the following items are present in the figure legend, table legend, main text, or Methods section.

n/a	Confirmed	
☐	☒	The exact sample size (*n*) for each experimental group/condition, given as a discrete number and unit of measurement
☐	☒	A statement on whether measurements were taken from distinct samples or whether the same sample was measured repeatedly
☐	☒	The statistical test(s) used AND whether they are one- or two-sided<br>*Only common tests should be described solely by name; describe more complex techniques in the Methods section.*
☒	☐	A description of all covariates tested
☒	☐	A description of any assumptions or corrections, such as tests of normality and adjustment for multiple comparisons
☐	☒	A full description of the statistical parameters including central tendency (e.g. means) or other basic estimates (e.g. regression coefficient) AND variation (e.g. standard deviation) or associated estimates of uncertainty (e.g. confidence intervals)
☐	☒	For null hypothesis testing, the test statistic (e.g. *F*, *t*, *r*) with confidence intervals, effect sizes, degrees of freedom and *P* value noted<br>*Give P values as exact values whenever suitable.*
☒	☐	For Bayesian analysis, information on the choice of priors and Markov chain Monte Carlo settings
☒	☐	For hierarchical and complex designs, identification of the appropriate level for tests and full reporting of outcomes
☒	☐	Estimates of effect sizes (e.g. Cohen's *d*, Pearson's *r*), indicating how they were calculated

Our web collection on statistics for biologists contains articles on many of the points above.

Software and code

Policy information about availability of computer code

Data collection	Microscopic images were collected by an Olympus X63 fluorescence microscope associated with the Olympus CellSense software, western blots were recorded with ChemiDocTouch System (BioRad).
Data analysis	Fluorescent microscope images were analysed by open source Fiji /ImageJ software, western blots by BioRad ImagerLab software. Statistical analysis was performed with GraphPad v8.3. and Matlab R2012b. Code for simulation analysis was deposited in Github https://github.com/DidiCi/MMsimulation.

For manuscripts utilizing custom algorithms or software that are central to the research but not yet described in published literature, software must be made available to editors and reviewers. We strongly encourage code deposition in a community repository (e.g. GitHub). See the Nature Portfolio guidelines for submitting code & software for further information.

Data

Policy information about availability of data

All manuscripts must include a data availability statement. This statement should provide the following information, where applicable:

- Accession codes, unique identifiers, or web links for publicly available datasets
- A description of any restrictions on data availability
- For clinical datasets or third party data, please ensure that the statement adheres to our policy

This statement has been included : All data are included in this article and its supplementary information files. Numerical source data for all graphs and charts are

provided in the Supplementary Data file. Uncropped Western blot images are presented in Supplementary Fig. 8. Other information about this study are available from the corresponding author upon reasonable request.

Human research participants

Policy information about studies involving human research participants and Sex and Gender in Research.

Reporting on sex and gender	NA
Population characteristics	NA
Recruitment	NA
Ethics oversight	NA

Note that full information on the approval of the study protocol must also be provided in the manuscript.

Field-specific reporting

Please select the one below that is the best fit for your research. If you are not sure, read the appropriate sections before making your selection.

☒ Life sciences ☐ Behavioural & social sciences ☐ Ecological, evolutionary & environmental sciences

For a reference copy of the document with all sections, see nature.com/documents/nr-reporting-summary-flat.pdf

Life sciences study design

All studies must disclose on these points even when the disclosure is negative.

Sample size	No statistical methods were used to determine sample size
Data exclusions	No data were excluded.
Replication	Experiments have been conducted in at least 2 replicates or more, indicated in the legend.
Randomization	No randomization was applied.
Blinding	No formal blinding was applied.

Behavioural & social sciences study design

All studies must disclose on these points even when the disclosure is negative.

Study description	NA
Research sample	NA
Sampling strategy	NA
Data collection	NA
Timing	NA
Data exclusions	NA
Non-participation	NA
Randomization	NA

Ecological, evolutionary & environmental sciences study design

All studies must disclose on these points even when the disclosure is negative.

Study description	NA
Research sample	NA
Sampling strategy	NA
Data collection	NA
Timing and spatial scale	NA
Data exclusions	NA
Reproducibility	NA
Randomization	NA
Blinding	NA

Did the study involve field work? ☐ Yes ☒ No

Reporting for specific materials, systems and methods

We require information from authors about some types of materials, experimental systems and methods used in many studies. Here, indicate whether each material, system or method listed is relevant to your study. If you are not sure if a list item applies to your research, read the appropriate section before selecting a response.

Materials & experimental systems

n/a	Involved in the study	
☐	☒	Antibodies
☒	☐	Eukaryotic cell lines
☒	☐	Palaeontology and archaeology
☐	☒	Animals and other organisms
☒	☐	Clinical data
☒	☐	Dual use research of concern

Methods

n/a	Involved in the study	
☒	☐	ChIP-seq
☒	☐	Flow cytometry
☒	☐	MRI-based neuroimaging

Antibodies

Antibodies used

Rabbit Anti-Xenopus Rif1 (Covalab, Villeurbanne, France; 1:500 dilution for immunofluorescence and 1:1000 dilution for WB)
Rabbit Anti-Xenopus Treslin (validated by W. G. Dunphy; 1:1000 dilution for WB)
Rabbit Anti-Xenopus MTBP (validated by W. G. Dunphy; 1:1000 dilution for WB)
Rabbit Anti-human MCM4 (Bethyl Cat# A300-193A, RRID: AB_162720; 1:2000 dilution for WB)
Rabbit Anti-Xenopus Drf1 (validated W. G. Dunphy; 1:1000 dilution for WB)
Rabbit Anti-Xenopus Ccd7 (validated and provided by J. C. Walter, Boston, USA; 1:1000 dilution for WB)
Rabbit Anti-Xenopus Cdc45 (validated W. G. Dunphy; 1:500 dilution for WB)
Rabbit Anti-Xenopus RecQ4 (validated W. G. Dunphy; 1:1000 dilution for WB)
Rabbit Anti-Xenopus TopBP1 (validated W. G. Dunphy; 1:1000 dilution for WB)
Rabbit Anti-Xenopus Orc2 (validated by J. L. Maller, University of Colorado and provided by EXRC, Portsmouth, UK, 1:1000 dilution for WB)
Rabbit Anti-Xenopus Orc1 (validated and provided by R. A. Laskey, Cambridge, UK; 1:1000 dilution for WB)
Rabbit Anti-Xenopus MCM7 (validated and provided by R. A. Laskey, Cambridge, UK; 1:1000 dilution for WB)
Mouse Anti-α Tubulin (Sigma Cat# T5168; RRID: AB_477579; 1:10000 dilution for WB)
Mouse Anti-human ssDNA (DSHB Cat# autoanti-ssDNA, RRID: AB_10805144; 1:100 dilution for DNA combing)
Rabbit anti-mouse AlexaFluor488 (Invitrogen Cat# A11059; RRID: AB_2534106; 1:50 dilution for DNA combing)
Goat anti-rabbit AlexaFluor488 (Thermo Fisher Scientific Cat# A11008; RRID: AB_143165; 1:200 dilution for immunofluorescence and 1:50 dilution for DNA combing)
Mouse Anti-streptavidin biotinylated (Abcyss Cat# BA-0500; RRID: AB_2336221; 1:50 dilution for DNA combing)
AlexaFluor594 conjugated streptavidin (Invitrogen Cat# S11227; 1:50 dilution for DNA combing)
Goat anti-mouse IgG HRP (Sigma-Aldrich Cat# A4416; RRID: AB_258167; 1:10000 dilution for WB)

	Donkey anti-rabbit IgG HRP (GE Healthcare Cat# NA934; RRID: AB_772206; 1:10000 dilution for WB)
Validation	Antibodies were validated by the supplier or were checked in the lab by Western Blotting on egg extracts and/or recombinant proteins.<br>Rabbit Anti-Xenopus Rif1 (validated in 10.4161/cc.11.6.19636; 10.1093/n10.1093 /nar/gkab756; 10.7554/eLife.75741)<br>Rabbit Anti-Xenopus Treslin (validated in 10.1016/j.cell.2009.12.049)<br>Rabbit Anti-Xenopus MTBP (validated in 10.1091/mbc.E17-07-0448)<br>Rabbit Anti-Xenopus Drf1 (validated in 10.1074/jbc.M307144200)<br>Rabbit Anti-Xenopus Ccd7 (validated in 10.1074/jbc.M307144200)<br>Rabbit Anti-Xenopus Cdc45 (DOI: 10.1091/mbc.e05-07-0671)<br>Rabbit Anti-Xenopus RecQ4 (validated in 10.1016/j.cell.2005.05.015)<br>Rabbit Anti-Xenopus TopBP1 (validated in 10.1016/j.cell.2005.12.041)<br>Rabbit Anti-Xenopus Orc2 (validated in 10.1038/s41467-022-34779-4; 10.15252/embj.201796585)<br>Rabbit Anti-Xenopus Orc1 (validated in 10.1016/S0960-9822(96)00746-4)<br>Rabbit Anti-Xenopus MCM7 (validated in 10.1073/pnas. 93.19.10189)

Animals and other research organisms

Policy information about studies involving animals; ARRIVE guidelines recommended for reporting animal research, and Sex and Gender in Research

Laboratory animals	Xenopus laevis
Wild animals	The study does not involve wild animals.
Reporting on sex	NA
Field-collected samples	NA
Ethics oversight	All animal experiments have been carried out in accordance with the European Community Council Directive of 22 September 2010 (2010/63/EEC). All animal care and experimentation were conducted in accordance with institutional guidelines, under the institutional license C 91-471-102. The study protocols were approved by the institutional animal care committee CEEA #59 and received an authorization by the Direction Départementale de la Protection des Populations under the reference APAFIS#998-2015062510022908v2 for Xenopus experiments.

Note that full information on the approval of the study protocol must also be provided in the manuscript.
